# Versatile On‐Chip Programming of Circuit Hardware for Wearable and Implantable Biomedical Microdevices

**DOI:** 10.1002/advs.202306111

**Published:** 2023-10-30

**Authors:** Ah‐Hyoung Lee, Jihun Lee, Vincent Leung, Arto Nurmikko

**Affiliations:** ^1^ School of Engineering Brown University Providence RI 02912 USA; ^2^ Electrical and Computer Engineering Baylor University Waco TX 76798 USA; ^3^ Carney Institute for Brain Science Brown University Providence RI 02912 USA

**Keywords:** application‐specific integrated circuits, biomedical implant, focused ion beam, laser ablation, post‐CMOS processing, programmable microdevices

## Abstract

Wearable and implantable microscale electronic sensors have been developed for a range of biomedical applications. The sensors, typically millimeter size silicon microchips, are sought for multiple sensing functions but are severely constrained by size and power. To address these challenges, a hardware programmable application‐specific integrated circuit design is proposed and post‐process methodology is exemplified by the design of battery‐less wireless microchips. Specifically, both mixed‐signal and radio frequency circuits are designed by incorporating metal fuses and anti‐fuses on the top metal layer to enable programmability of any number of features in hardware of the system‐on‐chip (SoC) designs. This is accomplished in post‐foundry editing by combining laser ablation and focused ion beam processing. The programmability provided by the technique can significantly accelerate the SoC chip development process by enabling the exploration of multiple internal circuit parameters without the requirement of additional programming pads or extra power consumption. As examples, experimental results are described for sub‐millimeter size complementary metal‐oxide‐semiconductor microchips being developed for wireless electroencephalogram sensors and as implantable microstimulators for neural interfaces. The editing technique can be broadly applicable for miniaturized biomedical wearables and implants, opening up new possibilities for their expedited development and adoption in the field of smart healthcare.

## Graphical abstract

The study presents a programmable microchip design for biomedical applications, addressing challenges in developing highly integrated and miniaturized circuits. The programmability is demonstrated through permanent and reversible modifications of internal parameters, such as resonance and cutoff frequencies, biasing, supply voltage, and chip‐specific address. This approach enables efficient evaluation and optimizations of circuit design, accelerating the development process for biomedical microchips.

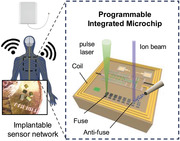

## 1 Introduction

Recent years have witnessed a surge in research focused on developing innovative biomedical devices.^[^ ^1^, ^2^, ^3^, ^4^, ^5^, ^6^, ^7^ ^]^ One example is the introduction of specialized wireless physiological monitoring devices suitable for neonatal and pediatric intensive‐care units.^[^ ^1^ ^]^ Related efforts have resulted in the development of implantable devices enabling continuous monitoring of vascular pressure, flow rate, and temperature.^[^ ^2^ ^]^ Progress has been made also in the field of wireless sweat or stress monitoring devices, featuring stretchability to enhance their utility in healthcare.^[^ ^3^, ^4^ ^]^ Elsewhere, there has been progress in the development of compact ingestible devices tailored for gastrointestinal monitoring as well as wireless optogenetic devices with rechargeable functionality introduced for biomedical research.^[^ ^6^, ^7^ ^]^

While wireless sensor devices are opening up new avenues for specific applications, there are strong incentives in general to further miniaturize their microelectronics with the aim to create minimally obtrusive and virtually invisible devices. Such wearable or implantable devices can significantly improve the patient experience or enable new healthcare capabilities in continuous monitoring of patients without interference to their daily lives. Ideally, any sensor device is an ultralow‐power monolithic chip which houses all the necessary electronic circuits including wireless telemetry. However, most approaches to date use individually packaged and power‐hungry commercial discrete components mounted onto printed circuit boards (PCBs) or equivalent substrates. For example, the devices based on PCB in Refs. [1, 2] have the size of several centimeters and consume up to tens of milliwatts.

Nonetheless, integration of circuit components into a single monolithic system‐on‐chip (SoC) microchip is possible if advanced semiconductor technologies are fully leveraged to develop application‐specific integrated circuits (ASICs) for specific functionality.^[^ ^8^, ^9^, ^10^ ^]^ The design of such chips offers the advantage of a significant reduction in size and power consumption compared to off‐the‐shelf circuit components, as shown in the Table S1 ^[^ ^11^ ^]^ (Supporting Information). An ultralow‐power design approach opens up also the possibility of power management by wireless means to eliminate the need for batteries which require frequent replacement and may pose additional safety concerns in cases of battery damage or electrolyte leakage.^[^ ^12^, ^13^, ^14^ ^]^ Furthermore, monolithically integrated biomedical microchips, as exemplified in Refs. [15, 16], offer an advantage in terms of biocompatibility. This is because voltage supply lines, which are electrically active and prone to breakage, are fully enclosed within the silicon die.^[^ ^17^ ^]^

There are several challenges in designing SoC circuits as microchips for biomedical devices. First, integrating all required features onto a single ASIC significantly increases the complexity of circuit designs, well beyond that of chips that rely on complementary off‐chip components. For autonomous operation, a single device must include power management, data processing, and communication in addition to the sensing or actuation circuits which in turn connect to tissue via a microelectrode interface.^[^ ^15^, ^16^, ^18^ ^]^ Second, quantitatively analyzing the performance of each separate functional block in a fully integrated wireless chip, an essential task for trouble‐shooting and/or fine tuning a chip during its development, is challenging as probes or wires near the radio frequency (RF) circuits can interfere with wireless links, leading to inaccurate assessments.^[^ ^19^ ^]^ Providing wired access to various bias reference nodes or E‐fuses through extraneous write‐in pads, which is a common technique for testing chips in a wired environment, is often impractical in wireless devices.

To address these challenges, we propose a novel hardware programming method for wearable and implantable wireless microchips for biomedical applications that incorporates metal fuses and/or anti‐fuses in the topmost metal layer of a complementary metal‐oxide‐semiconductor (CMOS) chip. The method offers a broadly applicable means for in situ tuning and optimizing any number of circuit functions within microimplants. Examples in this paper encompass front‐end mixed signal circuits for electrophysiology, those for RF resonance matching in remote energy harvesting and optimizing on‐chip power management. As such, the applicability of this method extends beyond a single circuit manipulation such as in microchip identification.^[^ ^15^ ^]^ Our toolkit employs laser ablation and focused ion beam (FIB) processing for in situ editing of CMOS chips of millimeter size, at sub‐micrometer resolution. By selectively ablating a metal fuse using pulsed laser or by shorting an anti‐fuse with ion‐beam assisted metal deposition and ion milling, we can explore, program, and optimize various internal chip parameters, greatly accelerating the biomedical SoC chip development process. This hardware programmability enables non‐volatile but reversible modification of the chip's internal parameters in post‐processing.

The use of fuse, FIB, and laser ablation techniques is commonplace in the industry to edit CMOS chips toward an acceptable prototype for subsequent mass production.^[^ ^20^, ^21^, ^22^, ^23^, ^24^ ^]^ However, by far, the major focus has been on one‐time programmable memory arrays.^[^ ^25^, ^26^ ^]^ There are a only handful of reports on the fine‐tuning of mixed‐signal circuits, chiefly to improve the precision of the circuits.^[^ ^27^, ^28^, ^29^ ^]^ More importantly and for broader applicability, what is lacking in the literature is the application of the laser ablation‐focused ion beam to in situ editing of the kind of complexity presented by mixed‐signal system‐on‐chip circuits targeting fully wireless wearable and implantable biomedical devices—where low‐power and low‐area design are of utmost importance. Also, one of the key differentiating factors of our method lies in the co‐design approach which enables sophisticated and systemic functional changes for biomedical sensing or stimulation based on hardware modifications in the post‐CMOS microfabrication process. The co‐design aspect in this study ensures a seamless integration in the circle of circuit design, structure, and its editing to enable both hardware programming and alterations to circuits. To the best of our knowledge, our work represents the first practical demonstration in utilizing fuse and anti‐fuse technologies, extending beyond mere identification, for programming sub‐millimeter size wireless microchips in biomedical SoC research.

To demonstrate the effectiveness of our approach, we describe in this paper the evaluation and optimization of subcircuits for three types of wireless microchips, each targeting a particular biomedical applications: i) electroencephalogram (EEG) brain recording microsensors, ii) microstimulators that inject current pulses for use in neural interfaces, and iii) communication chips designed to enable networks composed of many autonomous on‐ or in‐body sensor/actuators to send data simultaneously to an external receiver.

We have successfully optimized the performance of specific circuit functions by in situ editing such as the analog front‐end circuits for biosignal recording and circuits that generate unique chip‐specific addresses for wireless communication. The editing is also shown to facilitate the analysis and adjustment of on‐chip antenna RF resonances in various capacitance configurations, and has enabled the testing and optimizing a chip's regulated supply voltage. A key advantage in our approach is that fuse‐based circuit programming requires a minimal footprint and does not require an additional dedicated digital engine for programming, for example. This stands in contrast to conventional memory devices or E‐fuses, which typically rely on programming pads or dedicated digital engines.^[^ ^30^, ^31^ ^]^ The proposed fuse/anti‐fuse method does have the drawback of not enabling real‐time chip programming during active operation and necessitates a relatively slower, serialized post‐fabrication process while methods such as programming through wireless downlink communication can offer quicker alternatives. (We note that there are number of companies developing next generations of laser‐ion‐beam process tools for chip post‐process editing). However, in contrast to downlink programming, fuse/anti‐fuse programming possesses a unique advantage due to its capability for permanent yet reversible hardware modifications, which eliminates the need for repetitive microchip programming and added circuitry. A comprehensive explanation is available in Note 1 (Supporting Information). Furthermore, this method can be applied to chips of various sizes, ranging from a few hundred micrometers to millimeters, typically chosen in multi‐project wafer processes. This contrasts with conventional techniques like lithography, which face significant challenges when applied to such microscale dies. Last, while the examples below focus on wireless neural sensors and stimulators, the proposed hardware programmable design ideas can be extended quite generally to other functional integrated miniaturized microchips in broader biomedical use.

## 2 Results and Discussion

### 2.1 Approach to On‐Chip Programming of Hardware for Fuse‐Based Microsensors

**Figure 1:**
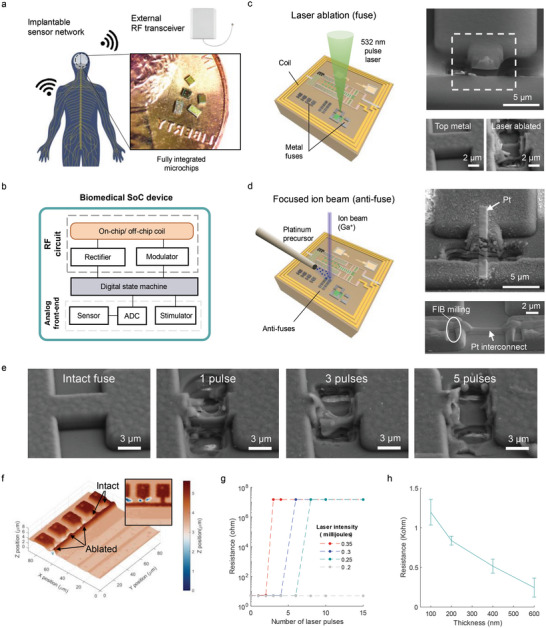
Programmable biomedical microdevices featuring fuse‐based co‐design for on‐chip editing of circuits for performance optimization. a) Concept schematic of wireless biomedical microdevices which communicate with and harvest energy from an external RF transceiver. The photograph shows the dimensions of fully integrated, programmable microchips designed in this study. b) Block diagram of the biomedical SoC microdevice, consisting of an RF circuit for energy harvesting and communication, a digital engine for data processing, and an analog front‐end for physiological sensing and electrical stimulation of target tissue. c) Schematic illustration of the in situ chip post‐process programming techniques using short pulsed lasers for precise etching of traces connected to the internal circuitry (left). SEM images of an aluminum metal fuse trace before and after laser ablation (right). d) FIB‐based anti‐fuse method, where a platinum (Pt) layer is selectively deposited on physically isolated nodes to create an electrical connection (left). SEM images show examples of Pt interconnection for a fuse already ablated by laser pulses (right top) as well as two top metal nodes exposed by FIB milling (right bottom). e) SEM micrographs of intact and laser‐ablated fuses, indicating disconnection of a 1.5 µm thick top metal trace after five consecutive laser pulses. The ablation feature size is adjustable to the target size, in this case, to 5 µm × 10 µm. f) 3D profiles of on‐chip microstructures after selective etching of three target traces by laser ablation. g) Measured resistance of a test structure with metallic traces and test pads, as a function of the number of laser pulse repetitions and laser intensity. Once the fuse is cut, the resistance is limited by the leakage current across electrostatic discharge diodes located on the test pads. h) Resistance of fabricated Pt interconnect lines (width: 1 µm, length: 20 µm) as a function of line thickness. The error bar represents the standard deviation (*n* = 3).

As the vehicle to develop and test the programmable co‐design ideas for post‐process editing of CMOS microchips for wearable/implantable biomedical applications (**Figure** **1**a), we leveraged our previous work on sub‐mm size low‐power ASICs for wireless neural interfaces.^[^ ^15^, ^32^, ^33^, ^34^ ^]^ The chip platform (here using the TSMC 65 nm RF process node) incorporates sensor or stimulator mixed‐signal front‐ends that interface with biological tissue, and digital state machines for data processing as well as programming current stimulation of target tissue. For wireless communication, the silicon die also includes an on‐chip RF coil, rectifier, and modulator for both RF signals and energy harvesting (Figure 1b). The multiple functional blocks are integrated as one standalone SoC microchip, for example, for minimally obtrusive neural signal recording or stimulation. In the co‐design approach, we designed, microfabricated, and then post‐process edited a series of chips by manipulating various combinations of arrays of fuse and anti‐fuse based metallic interconnects to optimize the performance of particular microchips. The chip circuit programming involved any number of functions, such as the mixed‐signal front‐end, the assignment of unique addresses across a population of individual chips, analysis and tuning of their LC resonances for maximum inductive coupling for wireless energy harvesting and signal transmission, and a means to control the chip global supply voltage, respectively. In the sections below, we describe wireless circuit optimization and characterization through the microfabrication process flow, which are rather challenging tasks for conventional wired or wireless chip design approaches.

Our microchips feature post‐process programmable fuses and anti‐fuses designed into the chip layout in the topmost aluminum metallization layer, which are subsequently manipulated using laser ablation and FIB processing of the die as received from the foundry. By selectively ablating metal fuses with a focused pulse laser (Figure 1c, left), we can cut the connection between any two electrical nodes as shown in the scanning electron microscope (SEM) image in Figure 1c (right top). The other SEM images (Figure 1c, right bottom, and Figure 1e) show how the selective ablation process can precisely remove the aluminum of the fuse with sub‐micrometer features preserved. The laser ablation process involves two steps: first, we open the top dielectric protective passivation layer (silicon nitride and oxide) using an UV laser pulse with a wavelength of 355 nm as shown in Figure S1 (Supporting Information); and then proceed to cut the top aluminum trace using a green 532 nm laser. Figure S2 (Supporting Information) provides a complementary cross‐sectional view of the ablated fuse, confirming the applicability of laser ablation to the smallest size allowed by the circuit design rule of the selected semiconductor process (3 µm × 3 µm for the TSMC 65 nm node). As another example, connecting two separate electrical nodes can be accomplished by applying a FIB processing tool on an anti‐fuse structure, as depicted in Figure 1d. The SEM images on the right display an ion‐beam assisted deposition of a thin platinum (Pt) film to form a shorted connection for either the ablated fuse (top) or the anti‐fuse (bottom). This method allows us to program and edit connections in open fuse‐laden circuits after CMOS fabrication and ablated fuses to selectively reverse the laser cutting.

The 3D profiling image in Figure 1f also illustrates how laser ablation selectively targets the fuse without damaging nearby circuit elements such as the 3‐turn inductive near‐field antenna coil, while up to 4 µm etching can be reached into the substrate at the fuse. To prevent unintended circuit damage, we typically positioned the fuse on areas where there are no underlying circuits (TSMC 65 nm RF process node has some 10 metallization layers, the active transistor layers are deep in the structure). Figure 1g shows resistance changes based on repeated laser pulse shots and the pulse energy for a laser beam area of 12 µm × 6 µm. The plot demonstrates that at least 0.25 mJ of laser pulse energy is required to cut the aluminum and that a stronger pulse can achieve this with fewer repetitions. For the work in this paper, we selected 0.25 mJ We note that, once the fuse is cut, the resistance is limited by the leakage current flowing across the electrostatic discharge diode on the chip's test pads. Consequently, the actual resistance of the ablated fuse is expected to be significantly higher, well beyond tens of Megaohms. As for the FIB processing, the example of Figure 1h compares the resistance of anti‐fuse based on the thickness of the FIB deposited Pt layer, with thicker layers resulting in lower resistance, consistent with previous findings.^[^ ^35^ ^]^ Still, in the circuit application examples shown below, these resistances can be considered sufficiently low not to affect the functional outcome, although there may be circumstances where it may be desirable to deposit an even thicker layer for lowering the resistance.

### 2.2 Co‐Design for Hardware Programming of a Neural Signal Amplifier

**Figure 2:**
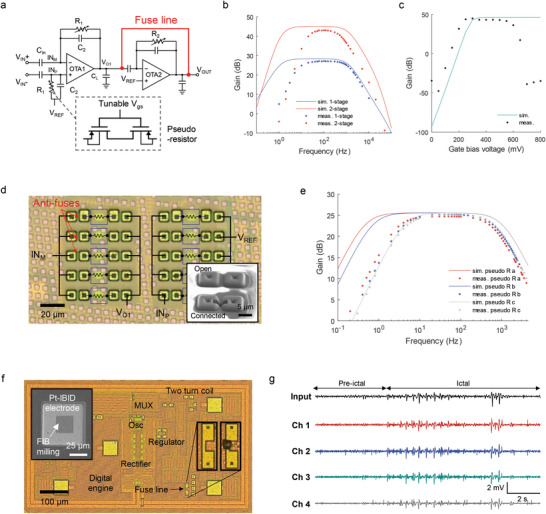
Evaluation and optimization of wired and wireless neural sensors incorporating pseudo‐resistors. a) Schematic of a two‐stage low‐noise capacitively coupled neural amplifier with a gate‐bias voltage‐controlled pseudo‐resistor (R_1_, R_2_) in a feedback loop. Each amplifier stage can be selectively tested by laser ablating the embedded fuse line (red), shorting the second stage amplifier input to output. b) Frequency responses of single‐ and two‐stage amplifiers, obtained through measurement and simulation. c) Gain of the two‐stage amplifier in response to a 100 Hz input signal as a function of the gate bias voltage (simulation and measurement). d) Photograph of an array of pseudo‐resistors and anti‐fuses, along with the state status of their connectivity to the OTA. The SEM image shows an original anti‐fuse and a connected fuse after FIB milling and Pt deposition. e) Gain and bandwidth of three versions of single‐stage amplifiers with different pairs of pseudo‐resistors, both simulated and measured. Additional details on the pseudo‐resistors are provided in Table S1 (Supporting Information). f) Wireless four‐channel EEG recording chip incorporating fuses to edit and control multiple features: pseudo‐resistor bias voltage, regulator, oscillator, and other components. The chip has dimensions of 500 µm × 800 µm. Inset in (f) shows Pt‐IBID (ion‐beam induced deposition) sensing electrodes fabricated on the wireless microchip using FIB after first milling to remove a thin aluminum oxide layer. g) Four‐channel recording of the wireless EEG microchip capturing proxy epilepsy pre‐ictal and ictal waves injected into saline. Abbreviations: sim.: simulated., meas.: measured.

Biosensing neural amplifiers typically consist of two or three preamplifier stages to achieve adequate bandpass response and large gain.^[^ ^36^, ^37^, ^38^ ^]^ In our design, we chose a widely employed two‐stage neural amplifier configuration that incorporates high resistance pseudo‐resistors in the G*Ω* range to amplify low‐frequency signals while canceling DC offset from the electrode‐electrolyte interface.^[^ ^39^ ^]^ A pseudo‐resistor is a circuit component that emulates the behavior of a high‐resistance resistor and is typically constructed using PMOS transistors to achieve a high resistance per unit area.^[^ ^40^, ^41^ ^]^ However, to accurately simulate the small leakage current across one or more pseudo‐resistor can be challenging, necessitating extensive testing and analysis of the fabricated chip in an attempt to quantify the amplifier's performance.^[^ ^42^, ^43^ ^]^ As an illustration of our chip editing approach, we chose an application of a microchip neural sensor for EEG brain signal recording. We first fabricated wired versions of the chip's amplifier (**Figure** **2**a) before proceeding to build a fully wireless EEG sensor chip. In this design, the gain of the first‐stage amplifier (*A_M_*) is given by *C_in_*/*C*_2_, the low cut‐off frequency is determined by 1/(2π*R*_1_ *C*_2_), and the high cut‐off frequency is defined as *G_m_*/(2π*C_L_A_M_*), where *G_m_* is transconductance of the first operational transconductance amplifier (OTA), and *f* is the frequency. The same set of equations applies to determine the parameters of the second amplifier as well.^[^ ^39^ ^]^ We designed the neural amplifier circuit by incorporating fuse features to pseudo‐resistors, which consisted of serial‐connected PMOS devices, and a small capacitor (C_2_) that provides a feedback path.^[^ ^44^, ^45^ ^]^

Due to the very large resistance of the pseudo‐resistors, transistor‐based switch circuits are not useful for modifying the amplifier's feedback loop. The fuses approach overcomes that problem in that a significantly higher resistance results when a fuse line (Figure 2a) is properly ablated. Measurement results shown in Figure 2b display the gain and bandwidth of the single‐stage and two‐stage amplifiers, where the fuse technique enables their separate characterization. The measured low cutoff frequency for the single and two‐stage amplifiers was ≈8 and 20 Hz, respectively, while the simulated values were 1.12 and 1.75 Hz. The measured gain of the two‐stage amplifier was 45.02 dB, which is 2.05 dB lower than the simulated values, potentially due to process variance affecting the small capacitance (C_2_) in the design.

Our design also incorporated a similarly tunable pseudo‐resistor to control the low cutoff frequency of the amplifier by adjusting the gain bias voltage,^[^ ^44^, ^45^ ^]^ as shown in Figure S3 (Supporting Information). Figure 2c summarizes the simulated and measured gain of this amplifier at 100 Hz, as a function of the gain bias voltage (*V*_gs_), showing that the amplifier achieved the nominal gain around half of the supplied voltage. Notably, when the gate bias voltage exceeded 600 mV, the amplifier exhibited significantly suppressed gain, potentially attributed to the high transient time caused by the large resistance of the pseudo‐resistor. These results confirm the usually encountered challenges in trying to model the small leakage currents across pseudo‐resistors. Here we show the effectiveness of our fuse method in providing a window to a systematic analysis of the amplifier and the usefulness of on‐chip editing to adjust the design, for example, gate bias voltage, using fuses in wireless chips.

At the full chip level, our hardware programming method offers the flexibility to select different combinations of pseudo‐resistors with an array of anti‐fuses. Figure 2d illustrates an implemented ASIC design with five examples of pseudo‐resistor pairs, connected to the neural amplifier circuit described in Figure 2a (Figure S4, Supporting Information provides the overall chip layout with 25 types of pseudo‐resistors). The inset of Figure 2d shows the SEM image of an anti‐fuse in its original open state and connected state. The connection was achieved by etching the passivation layer and then depositing Pt between two anti‐fuse points. We implemented three versions of single‐stage amplifiers with different pseudo‐resistor designs and measured their gain and bandwidth, as depicted in Figure 2e. Further details on the three pseudo‐resistor designs are provided in Figure S5 and Table S2 (Supporting Information). While there were distinct differences in the simulated low‐cutoff frequency among the three amplifier designs, the measured values were in fact quite similar, with ≈2.5, 3.5, and 4.5 Hz for pseudo‐resistor types a, b, and c, respectively. This type of information provides a precise evaluation of the various pseudo‐resistor types and serves as a guide for designing the amplifier according to the frequency range of the target neural or other biosignals of interest. Selecting high resistance pseudo‐resistors in this manner is not feasible with other circuit‐based design/fabrication methods to the best of our knowledge. The idea is simple but effective in that, for example, our anti‐fuse approach enables the conversion of an open circuit to a connected low‐resistance wire.

Using the co‐design outcomes, we proceeded to design a fully wireless four‐channel prototype chip for recording EEG signals, with a focus on gamma band signals. Leveraging the wireless neural sensor backbone which we previously developed,^[^ ^15^, ^33^, ^34^ ^]^ we produced a 500 µm × 800 µm size chip with layout in Figure 2f. Multiple fuse lines were selectively ablated on the chips to control the gate bias voltage. Further, we employed the FIB maskless micropatterning capability to in situ integrate 60 µm × 60 µm thin film Pt sensing electrodes onto the chip's aluminum contact pads, with an impedance range suitable for neural recording,^[^ ^46^ ^]^ as illustrated in the inset of Figure 2f and Figure S6 (Supporting Information). In test recordings, we immersed the entire chip in saline while wirelessly transferring power (at 915 MHz). This neural sensor had a sampling rate of 250 Hz and a low cutoff frequency of 30 Hz, making it suitable for gamma band EEG signal recording. Figure 2g illustrates an example of the four‐channel recording from the chip capturing both pre‐ictal and ictal waveforms injected into saline by an external electrode, revealing a potential for detecting epileptic events. The proxy EEG datasets were acquired from Ref. [47]; details on the experimental setup and RF telemetry methods are described in the Experimental Section. This tunable EEG sensor design enables hardware‐level optimization of amplifier characteristics based on the epileptic waveform of the specific patient, eliminating the need for further programming during the operation (See Note 1, Supporting Information).

### 2.3 Encoding a Unique Identifier for Wireless Control of a Target Microstimulator Chip

**Figure 3:**
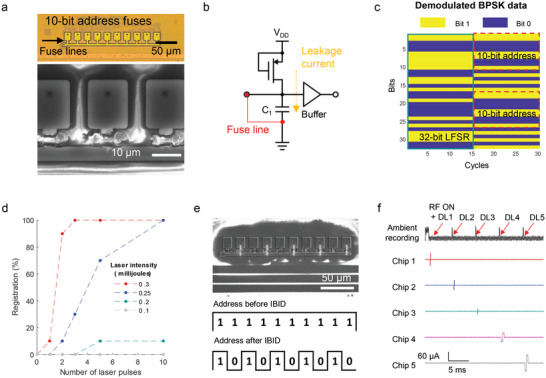
Current injecting microstimulator chip with programmable metal fuse address. a) The optical image and SEM image reveal the 10‐bit fuse lines before and after laser ablation. b) A schematic of a circuit for the detection of fuse line states and the generation of binary bit output (0 for short and 1 for open) with ultra‐small leakage current (≈pA). c) RF backscattered 960‐bit from the wireless microchip (near 945 MHz) showing a 32‐bit predefined LFSR sequence and a 10‐bit registered address on the digital engine. d) Percentage of address bits changing from 0 (short) to 1 (open) as a function of laser intensity and number of laser pulses (*n* = 10). e) Reverse programming of the ablated open fuse achieved using Pt‐IBID. The SEM image at the top shows selectively deposited Pt traces on the ablated fuses, while the bottom traces depict the corresponding address bits before and after IBID. f) Pulsed current stimulation generated by five different chips utilizing the unique address assigned to each chip. Each chip responds to the downlink (DL) command containing the address information and generates programmed current stimulation at an assigned time slot, demonstrating how the channel selectivity achieved through the 10‐bit address fuses.

One presently investigated approach to modulating physiological circuits such as those in the brain and the heart is the use of multiple electrode sites for patterned current stimulation. One of our proposed distributed microimplant scenarios involves a wireless microstimulator chip. For a network of such microdevices, it is essential that each chip has a unique identifier. Expanding on the work in Ref. [15], we have designed a 10‐bit programmable fuse address as co‐design with the essential core circuits as depicted in **Figure** **3**a. The enlarged fuse geometry and SEM image provide a closer view at the fuses ablated using a 532 nm pulse laser. As received from the foundry, these fuse lines were connected to the ground in parallel to charging capacitors (C_1_), resulting in an output bit value of zero.^[^ ^32^, ^48^ ^]^ However, after the fuses are ablated, a transistor linked to the voltage supply generates a minute leakage current (150 pA) which charges up C_1_ and flips the bit to one (Figure 3b). Careful consideration was given to the time constant for capacitor charging; here we chose 9.27 µs. This choice ensures that the power‐on‐reset triggers the digital engine to read out the address after the charging process is complete. This circuitry allows for the assignment of a unique address to each chip, while consuming minimal current.

The current delivering microchip incorporates two digital engines, one of which is dedicated to generating a backscattering signal upon chip activation. The backscattering signal consists of a 960‐bit sequence, including a repeating 32‐bit linear‐feedback shift register (LFSR) sequence and a repetitive 10‐bit address, as illustrated in Figure 3c. The start‐up backscattering block allows us to verify whether the laser‐programmed address is correctly recognized by the digital engines. Figure 3d presents the percentage of registered address changes (“zero”: short to “one”: open) based on the laser intensity and the number of laser pulses. The results demonstrate that, as already discussed, a laser intensity greater than 0.25 mJ is required for reliable ablation of the metal fuses. Once the fuses are ablated, each can be treated as an anti‐fuse, enabling the use of FIB to deposit metal layers to reverse the connection between any two anti‐fuse terminals. Figure 3e demonstrates how, following the deposition of 200 nm metal layers, the chip successfully recognizes the connection with a remanent resistance of ≈850 Ω. Importantly, this type of reversible programming capability of the metal fuse and the proposed circuit is quite reliable and has the benefit of a small footprint and very low current consumption.

A second digital engine within the microstimulator chip performs the demodulation of the remotely transmitted RF amplitude‐shift keying pulse width modulation (ASK‐PWM).^[^ ^33^, ^49^ ^]^ The ASK‐PWM downlink is transmitted from the external RF hub by temporal modulation of the transmitting (Tx) power. The on‐chip ASK‐PWM demodulator compares the high and low Tx energy states to reliably identify the downlink bit, maintaining a data rate of 1 Msps independent of the on‐chip clock frequency which may vary across a chip population. Once the downlink is demodulated, the digital command sequence sent by the remote command center (via Tx) can be recovered so that each chip can utilize its unique address to execute the command specific to that chip. Figure 3f illustrates the case for five wireless stimulator chips, where each chip with its unique fuse address has been programmed to deliver electrical stimulation by the RF downlink signal. For the downlink command packet, we activate one chip at a time in a daisy chain sequence to generate a stimulation waveform with discrete current amplitudes (here 50 or 100 µA peak‐to‐peak) and pulse widths (100 µs and 1 ms). In our prior research, we utilized physically unclonable functions (PUFs) as unique chip addresses^[^ ^50^, ^51^ ^]^ which have a smaller size compared to physical metal fuses on‐chip. However, process variable‐based PUF addresses were susceptible to environmental fluctuations and occasionally resulted in random changes to the address bits.^[^ ^52^, ^53^ ^]^ By contrast, the hardware‐encoded fuse address presented here provides a robust, unique address to each chip, scalable to potentially thousands of error‐free identifiers.

### 2.4 Programming Microchips for Efficient RF Wireless Energy Harvesting

In battery‐less wireless RF devices, energy harvesting plays a crucial role as it provides power to the subsequent circuits. Additionally, wireless energy harvesting is closely tied to safety concerns, as low‐efficiency systems require higher Tx power, which must comply with regulatory limits.^[^ ^54^, ^55^ ^]^ However, conducting a systemic analysis of wireless transfer efficiency in small devices,^[^ ^18^, ^19^, ^56^ ^]^ ranging from a few millimeters to sub‐millimeters in size (Figure 2a), poses challenges. Off‐chip probes, wires, or coaxial connectors commonly used for the characterization of RF wireless links can distort the electromagnetic field whether in the near or far field. As alternative methods, projecting energy such as converting RF first to light^[^ ^19^ ^]^ has been employed to evaluate efficiency. Here, using the clock frequency of the on‐chip oscillator as a wireless power indicator, we applied the fuse‐based editing to assess and tune its energy harvesting efficiency in the near field (inductively coupled) RF regime.

**Figure 4:**
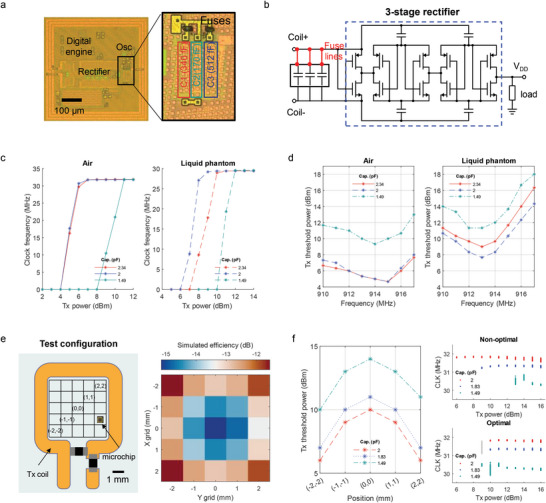
Optimizing wireless inductive energy harvesting using a prototype chip with laser‐programmable fuses. a) Microphotograph of the prototype chip featuring fuse‐connected capacitors for resonance RF tuning, a rectifier, and a digital engine. b) Schematic of the 3‐stage cross‐coupled rectifier and capacitors. c) Graphs depicting the average clock frequency recovered via BPSK demodulation, showing the effect of external Tx power source levels (at 915 MHz) and tuning capacitances in both air and liquid head phantom environments respectively (*n* = 3). d) Plot illustrating the relationship between the threshold Tx power for chip activation as a function of the incident Tx tone frequency and matching capacitance. e) Illustration of the Tx coil and microchip's location in relation to the Tx coil (left) and simulation results of wireless efficiency in various positions (right). f) Measured Tx threshold power level depending on the location of the microchip and tuning capacitance (left). Dependence of clock frequencies on the Tx power for three microchips, shown in two configurations: non‐optimal (right top) and optimal (right bottom), indicating the efficiency improvement in the latter (packet number for data points = 200 for each condition, the legend shows matching capacitance value in each chip). Abbreviations: cap.: capacitance, CLK: clock frequency.

For evaluating the energy harvesting efficiency, it is crucial to have access to fine‐tuning the wireless link to identify and optimize the LC resonance frequency of each microchip, which can be used for future design iterations. To achieve this, we added fuse lines to a chip's harvesting circuit to test various capacitor values and identify the resonance point near 915 MHz by monitoring the RF transfer efficiency. **Figure** **4**a shows a microphotograph of the prototype chip developed to wirelessly assess the resonance point. The chip incorporates a rectifier, regulator, oscillator, digital engine, and modulator for generating binary phase‐shift keying (BPSK) backscattering. The circuit diagram in Figure 4b illustrates the configuration with capacitors across the microcoil and the modified 3‐stage cross‐coupled rectifier reproduced from our previous studies which achieved high rectifying efficiency even at low incoming RF power.^[57^ ^]^ The resonance of the system is determined by the combined impedances of the on‐chip coil, the matching capacitors and the impedance of the rectifier. We embedded fuse lines along the connections to the three parallel capacitors, each with a different capacitance value (170, 340, 512 fF).

For the experiment, we securely fixed the microchip within the external Tx coil, on a PCB (Figure S7, Supporting Information). This step was important in eliminating potential confounding factors, such as variations in the distance between the Tx coil and the on‐chip receiving microcoil as well as the influence of surrounding material permittivity. Subsequently, we measured the clock frequency of the chip at different Tx power levels, both in the air and when immersed into a liquid head phantom. The clock frequency was derived from the BPSK backscattering signal, as described in Ref. [15]. The liquid head phantom approximated the permittivity of head tissue, allowing the assessment of the surrounding media's impact on wireless transfer efficiency.^[^ ^54^, ^58^ ^]^

In the baseline measurement with a total parallel capacitance of 2.34 pF, we observed that the average threshold Tx power required to activate the chip was 6 dBm in the air and 10 dBm in the liquid phantom, as depicted in Figure 4c. Figure 4d shows the minimum required Tx power to operate the chip based on the Tx tone frequency. The results show that the required power level was slightly higher in the liquid phantom compared to the air. This is because the liquid phantom affected the resonance of the Tx coil and introduced additional path loss from absorption in the conductive media. Subsequently, we ablated the connection to the 340 fF capacitor while keeping all other conditions constant. The threshold Tx level remained the same in the air, but it decreased by 1.33 dB in the liquid phantom at the target frequency of 915 MHz. When ablating another capacitor, 512 fF, the overall efficiency significantly decreased, indicating that the resonance frequency of the wireless devices was beyond the test range. These findings give a snapshot of how the fuse‐tuning idea can enable a systematic adjustment of tuning capacitance and identification of specific resonance points in the target media.

In addition, the tuning capacitors can optimize power harvesting in a population of wireless devices. For a population of microsensors to gather information over a wide area (e.g., of the brain cortex), multiple devices need to form a network to collaboratively capture information from the target zone. In an inductively coupled regime, the size of the external Tx coil defines the target area, resulting in non‐homogeneous fields as illustrated in Figure 4e (left). Figure 4e (right) shows RF simulation results depicting how wireless transfer efficiency varies spatially across the area of the Tx coil. The simulation also reveals that microdevices closer to the perimeter of the Tx coil harvest more energy than others, a finding confirmed by the measurements (Figure 4f, left). We tested the Tx threshold power at various locations while adjusting the matching capacitors. The results show, for example, that if we place chips with 1.49 pF parallel capacitance near the perimeter of the Tx coil, they harvest a similar amount of energy as chips with 2 pF tuning capacitance positioned far away from the Tx coil's perimeter (i.e., in the center). To illustrate, we simultaneously activated three chips with different tuning capacitance while arranging them in the Tx coil in different configurations. Increasing the Tx power, we observed the activation of chips through RF backscattering, as indicated by Figure 4f (right), which shows the recovery of three distinct clock frequencies from the backscattering packets. In a non‐optimal chip arrangement, chips are turned on at different Tx power levels, requiring ≈13 dBm to activate all chips. However, in an optimal configuration, all chips were activated at ≈9 dBm, reducing the required Tx power level by 4 dB.

### 2.5 Programming the Supply Voltage for Optimal Wireless Chip Performance

**Figure 5:**
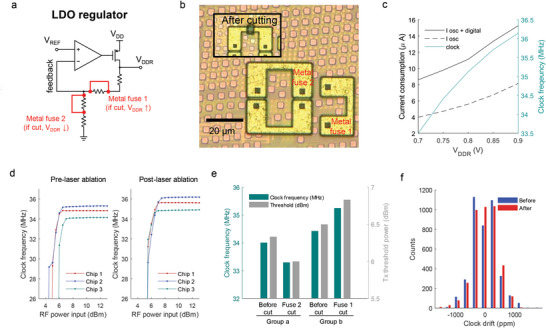
Control of chip voltage supply. a) LDO regulator featuring integrated metal fuses in the feedback loop for bypassing the resistors. b) Photographs of the top metal fuses before and after laser cutting; the embedded regulator lies underneath the metal fills. c) Circuit simulation demonstrating the current consumption of the clock oscillator and digital engine, as well as the clock frequency, all influenced by the regulated voltage supply. d) Variation in clock frequency of chips with respect to the Tx power level, before and after laser ablation of the fuse, illustrating the increase in average clock frequency and power requirement. e) Effects of cutting two different metal fuses (fuse 1 and fuse 2) on the clock frequency and threshold Tx power, indicating the possibility of increasing and decreasing the regulated voltage supply. f) Clock stability analysis from observing frequency drift over time before and after ablating the fuse 1 (packet *n* = 4000). Abbreviations: *V*_DDR_: regulated voltage supply, *V*_DD_: voltage supply, *V*_ref_: reference voltage, osc: oscillator, ppm: parts per million.

As the last example of chip editing, we show the utility of programmed access to the wireless microchips’ regulated supply voltage (*V*_DDR_). The V_DDR_ is a critical parameter that significantly impacts the functionality of the entire chip and affects a number of functional blocks such as the analog front‐end, the digital engines, and the on‐chip oscillator. To precisely control the *V*_DDR_, we have integrated fuses and serialized resistors into the feedback loop of the low‐dropout regulator (LDO)^[^ ^59^ ^]^ of our wireless EEG sensor as illustrated in **Figure** **5**a,b. By selectively ablating one of the two fuses, we can either step up or step down the feedback bias voltage, consequently influencing the supply voltage. As depicted in Figure 5c, the regulated supply voltage determines the clock frequency of the oscillator, ranging from 33.5 to 36.2 MHz, and influences the overall current consumption of the oscillator and the digital engine, which varies from 8.6 to 15.2 µA. Notably, higher supply voltages result in significantly higher energy consumption. However, it is important to note that the simulated performance of circuit blocks can be less accurate, particularly at lower voltages, which we employ, than the typical voltage (ideally 1.2 V in the 65 nm foundry process).^[^ ^60^ ^]^ Consequently, fabricating microchips with a fixed low supply voltage can lead to chip failure and poses a considerable risk.

Therefore, we have used the reversible laser programming technique to adjust the voltage supply for identifying the power‐saving configuration. In Figure 5d, we show the clock frequency in relation to the RF power input and the changes observed after ablating fuse 1 in the regulator circuit to increase the supply voltage. Although direct measurement of the supply voltage is not feasible in this particular chip, the increase in the clock frequency reflects indirectly the elevation in the supply voltage (from an average frequency 34.43 to 35.25 MHz). The required RF energy to activate the chip did show a slight average increase. Additionally, we investigated the impact of a lower supply voltage on the fuse configuration with results for both cases summarized in Figure 5e. Ablating another fuse (fuse 2) resulted in an average decrease of 0.7 MHz in the clock frequency and a decrease of 0.33 dBm in the required RF power level, indicating reduced power consumption on the chip. While not investigated further in this study, an additional fuse located on the oscillator itself can provide independent control over the clock frequency as well (details in Figure S8, Supporting Information).

In Figure 5f, we present the distribution of the clock frequency from 4000 collected packets over time, before and after the ablation of fuse 1. We observed a clock drift of ≈1000 ppm, which arises from the inherent instability of the relaxation oscillator chosen for its low area and power characteristics. Importantly, this clock drift remained consistent before and after the laser ablation, indicating that the programming of the voltage supply does not significantly impact clock stability. These results propose that the integration of fuses in the regulator enables the evaluation of systemic changes under various voltage supply conditions, thereby facilitating the development of microdevices with minimal power consumption.

## 3 Conclusion

This study has focused on a programmable co‐design approach for developing monolithic SoC microchips for wearable and implantable biomedical applications as an alternative to biomedical devices currently mostly realized as heterogeneous assemblies of off‐the‐shelf components. The wireless neural microsensors and stimulators described in this paper each incorporate a number of integrated circuit functions laid out on a sub‐millimeter size silicon die and designed to be remotely accessible either as individual devices or as large populations of autonomous sensors in a wireless network. To optimize microchip performance and to accelerate the development of minimally obtrusive and invasive healthcare‐related smart chips in general, we have introduced a simple yet potent co‐design idea which incorporates fuse and anti‐fuse structures into the ASIC design with minimal overhead. Through a combination of post‐process laser ablation and FIB patterning/thin films deposition, we have shown how to selectively disrupt or connect multiple electrical nodes at critical circuit points, enabling programmability and flexibility for tailoring a microchip's performance. The approach allows us to precisely modify the microchips' internal parameters, enhancing their functionality and adaptability, examples in this paper including the programmable co‐design of a biosensing neural amplifier and a four‐channel wireless EEG sensor chip. To ensure reliable wireless communication by a network of any type of microdevice with an external transceiver, we designed a chip incorporating a 10‐bit metal fuse address to write‐in a unique, secure identifier. In the domain of wireless energy harvesting and management, we applied laser ablation to improve the efficiency in an inductively coupled system. In terms of broader applicability, our work of the fuse‐based co‐design and chip hardware editing can be extended to other functional integrated microsensors and actuators, especially those for biomedical applications where sensor size and power are at a premium. The approach allows for a comprehensive exploration of the parameter space of preliminary chip designs prior to mass production or fine‐tuning of individual chips to meet specific requirements. Thus, the work presented here can help pave the way for the development of next‐generation wearable and implantable smart sensors, facilitating their widespread adoption and daily use in personalized healthcare.

## 4 Experimental Section

### Laser Ablation Process for Programming Microchip Hardware

The laser ablation process utilized a commercial pulsed laser cutting system (Ezlaze 3 cutting system, New Wave Research, equipped with a 100× objective). The wavelengths of 532 nm (green) and 355 nm UV were selected, respectively by the optical properties of the target layers used in the TSMC 65 nm LP RF CMOS foundry fabrication. The equipment offered a variable spot size and an optical attenuator for precise control of the delivered energy. Prior to initiating the ablation process, the silicon chips from the foundry were diced to separate individual millimeter or sub‐mm sized chips. The diced chips were attached to a glass slide using double‐side polyimide tape which allows for easy handling and retrieval of chips after the ablation step. The laser ablation procedure was carried out sequentially, starting with the UV laser to create a 12 µm × 6 µm opening in ≈1 µm‐thick dielectric silicon nitride/oxide protection/passivation topmost layer, which facilitated access to the top metal layer. This step involved using an average power of 0.25 mJ and applying five pulses. Subsequently, the green laser was used to cut the aluminum fuse trace, with a typical spot size of 12 µm × 6 µm. The parameters for power and the number of repetitions were adjusted according to the specific test scenarios under consideration. The resistance of the fuse lines was measured using a Keithley 2400 source meter, while the 3D surface profile image of the ablated fuse was obtained using the Taylor Hobson CCI HD Optical profiler.

### FIB Milling and IBID Tools for Programming the Anti‐Fuses

The anti‐fuse modification process involved the use of FIB milling and ion‐beam induced deposition (IBID) techniques. A fixed Ga ion energy of 30 keV was used, and the size of the target area dictated the ion beam current. Initially, the dielectric passivation layer was locally etched by FIB milling (FEI Helios NanoLab 600i DualBeam) to expose the aluminum layer. For the anti‐fuse dimensions in this paper, the etching dimensions were 6 µm × 2 µm, with a depth of 1.5 µm, achieved with ion beam current of 0.92 nA. Next, the desired line interconnection was created in the IBID process by deposition of a thin film of Pt. The deposited line had a slightly narrower width than the milled area, measuring 6 µm × 1 µm with a 200 nm thickness. The deposition rate was ≈0.013 µm^3^ s^−1^. As for the in situ deposition of the Pt microelectrodes on EEG sensor chips, the process began with FIB milling to remove the aluminum oxide layer in 25 µm × 25 µm area. Subsequently, a Pt layer, 60 µm × 60 µm × 0.3 µm was patterned overlying the chip's aluminum pads.

### Procedure for Designing and Testing Wireless Microchips

The wireless microchips which were co‐designed to house fuse and anti‐fuse structures were programmed by building upon the previous studies^[^ ^15^, ^32^, ^33^, ^34^ ^]^ in utilizing the TSMC 65 nm mixed‐signal/RF low‐power CMOS foundry process. All the microchips featured a modified three‐stage cross‐coupled rectifier in the energy harvesting block, but only the EEG recording chips and the data communication prototype chip included an LDO regulator. The on‐chip relaxation oscillator generated a nominal 30 MHz clock, and the digital engine operated at a nominal frequency of 10 MHz. To measure the resonance frequency wirelessly, a prototype chip that transmitted backscattering packets 50 times per second, each containing a unique 511‐bit digital code specific to the chip is utilized. Circuit simulation and design were conducted using Cadence IC618, and electromagnetic simulation was performed using Ansys HFSS. In the HFSS simulation, a head phantom model utilizing dielectric parameters sourced from Ref. [54] is employed.

Tests were conducted on the received silicon chips under a fully wireless testing environment using the previously developed wireless communication setup.^[^ ^15^ ^]^ To couple RF signals to wireless microchips, a FR4 (1.6 mm thickness) or polyimide PCB (0.1 mm thickness) was utilized, incorporating the single‐turn transmitter Tx coil (5 mm × 5 mm area, 3.5 µm thick copper) and an impedance matching capacitor network. As the RF transceiver and power source, a software‐defined radio (SDR), the “Raptor” model by Rincon Research, along with Analog Devices AD9361 transceiver chips and Zynq SoCs is employed. The SDR generated an RF baseband carrier at 915 MHz, which was amplified by an RF power amplifier (ADL5605‐EVALZ, Analog Devices). An RF surface acoustic wave duplexer (D5DA942M5K2S2, Taiyo Yuden) isolated the backscattered signals from the downlink carrier. The SDR performed signal amplification, downconversion from 945 MHz to DC, and analog‐to‐digital conversion at a rate of 30 MSa s^−1^ (12‐bit). The digitized IQ data were then transferred to a personal computer for BPSK data demodulation using MATLAB, following the details provided in Refs. [15, 61]. For testing in liquid phantom, the wireless chips were immersed in the respective liquids, with a small well used as a container (Figure S7, Supporting Information). The liquid phantom was composed of 64.81% 1,2‐propanediol, 0.79% NaCl, and 34.4% deionized water.^[^ ^54^ ^]^ For the EEG chip test, a benchtop setup is employed where the Tx coil, encapsulated by Polydimethylsiloxane (PDMS), was integrated at the bottom, alongside the saline well, and the microchip was positioned on top of the Tx coil. Following the infusion of saline into the well, the pre‐ictal and ictal EEG waveforms were delivered using Ag/AgCl electrodes near the microchip.

## Conflict of Interest

The authors declare no conflict of interest.

## Supporting information

Supporting Information

## Data Availability

The data that support the findings of this study are available from the corresponding author upon reasonable request.
