# Supplementary material for: Versatile On‐Chip Programming of Circuit Hardware for Wearable and Implantable Biomedical Microdevices
Source: Adv Sci (Weinh). 2023 Oct 30;10(36):2306111. doi: 10.1002/advs.202306111 (PMC10754128; doi:10.1002/advs.202306111)
Supplement: Supplementary file 1 — Supporting Information [file ADVS-10-2306111-s001.pdf]

ADVANCED SCIENCE
Open Access

Supporting Information

for *Adv. Sci.*, DOI 10.1002/advs.202306111

Versatile On-Chip Programming of Circuit Hardware for Wearable and Implantable Biomedical Microdevices

*Ah-Hyoung Lee, Jihun Lee, Vincent Leung and Arto Nurmikko**

Supporting Information

Versatile On-Chip Programming of Circuit Hardware for Wearable and Implantable Biomedical Microdevices

*Ah-Hyoung Lee†, Jihun Lee†, Vincent Leung, and Arto Nurmikko**
*Corresponding author. Email: arto_nurmikko@brown.edu
†These authors contributed equally to the work.

List of Figures and Tables

Table S1. Comparison between systems using discrete components and fully integrated systems
Supplementary Note 1. Comparison between downlink communication programming and fuse/anti-fuse hardware modification
Figure S1. Scanning electron microscope (SEM) image of the top metal fuse after ablating the dielectric passivation layer
Figure S2. SEM image and FIB cross-section image of the ablated top metal fuse
Figure S3. Low cutoff frequency of the single-stage amplifier as a function of the gate bias voltage
Figure S4. Circuit layout and connection geometry for anti-fuses, pseudo-resistors, and the neural signal amplifier
Figure S5. Circuit configuration of a pseudo-resistor evaluated using anti-fuse programming
Figure S6. FIB-based on-chip Pt microelectrode fabrication process
Figure S7. Arrangement for fully wireless chip testing
Figure S8. Integration of programmable fuses in relaxation oscillator subcircuits for on-chip clock frequency control
Table S2. Types and parameters for self-biased pseudo-resistors selected by anti-fuse programming

Table S1. Comparison between systems using discrete components and fully integrated systems

	[1]	[2]	[3]	[4]	[5]	[6]	**This work**
**Powering**	Battery	Wireless	RF wireless	RF wireless	RF wireless	RF wireless	RF wireless
**Size**	30 mm × 60 mm *	45 mm diameter	500 µm × 250 µm	1.5 mm × 1.4 mm	200 µm × 200 µm	500 µm × 500 µm	500 µm × 500 µm / 500 µm × 800 µm
**Type**	Physiological monitoring†	Blood hemodynamics monitoring‡	Neural recording	Neural recording	Neural stimulator	Neural recording/stimulation	Neural recording/stimulation
**Off-chip component**	All§	All§	None	Coil, Capacitor	None	None	None
**CMOS Technology**	N/A	N/A	65 nm CMOS	350 nm CMOS	130 nm CMOS	65 nm CMOS	65 nm CMOS
**Power supply (V)**	1.8/3.3	3.3	1.07	1.8	1.2	0.6-1	0.8-1
**Power consumption (µW)**	21000– 39600*	9200-42900*	10.5	< 300	8-38	< 30	< 30
**Telemetry**	Bluetooth	Bluetooth	OOK (Downlink), Miller encoded (uplink)	IR-UWB (Uplink) Single device	Passive downlink (Singe device)	BPSK (Multiple device Network)	BPSK (Multiple device Network)
**Tunability**	×	×	×	×	×	×	○

Abbreviation: OOK, On-off keying; IR-UWB, Infrared-ultra wideband; BPSK, Binary phase-shift keying.
† Heart rate, blood oxygenation, respiration rate, and temperature
‡ Blood pressure, flow rate, and temperature
§Antenna, battery, power management circuit, Bluetooth low energy (BLE) system-on-a-chip (SoC) and associated sensors.
***Estimated values based on the reference**

Supplementary Note 1. Comparison between wireless downlink communication programming and post-process fuse/anti-fuse hardware modification approaches

The utilization of downlink communication programming provides the advantage of adaptive and real-time adjustments for biomedical microchips. However, in any number of cases, the multiple limitations of downlink programming favor the utilization of the type of fuse programming described in this paper.

To begin, downlink programming predominantly employs transistor-based switches that exhibit low "short" state resistance and moderate "open" state resistance. Yet, these switches cannot deliver equivalent control as compared to the fuse method due to the inadequate resistance of the “open” state in the transistor-based switches. Figure 2 illustrates such a situation, wherein a high impedance pseudo-resistor cannot be effectively managed by a conventional transistor switch, thus demanding the incorporation of the fuse/anti-fuse control approach.

Furthermore, there is the "Cold start" problem. If the initial state of the circuit is not optimal, a fully wireless circuit might fail to initiate. In such instances, utilizing downlink communication to program the chip is infeasible. For instance, if the regulated VDD is designed to be exceptionally low, the chip may not initiate from the initial state, making it impossible for downlink communication to resolve the issue. On the other hand, adopting the fuse method to increase the regulated VDD permits a shift from the initial starting point to enable functionality even without chip activation (as depicted in Figure 5).

Additionally, downlink programming necessitates the integration of a downlink demodulator which can consume extra space and power. By contrast, in fuse programming no further wireless remote or electronic control is required as the hardware itself has already been modified thereby mitigating complexity and power consumption. Our wireless EEG sensor chips, for example, were designed to function without downlink communication given the benefits of fuse programming for hardware modification.

However, downlink programming holds an advantage over fuse programming in scenarios requiring real-time adaptive programming—a capability that fuse programming lacks. Hence, depending on the ultimate complexity of microchip design and its intended biomedical application, an implant system might benefit from accommodating both methods.

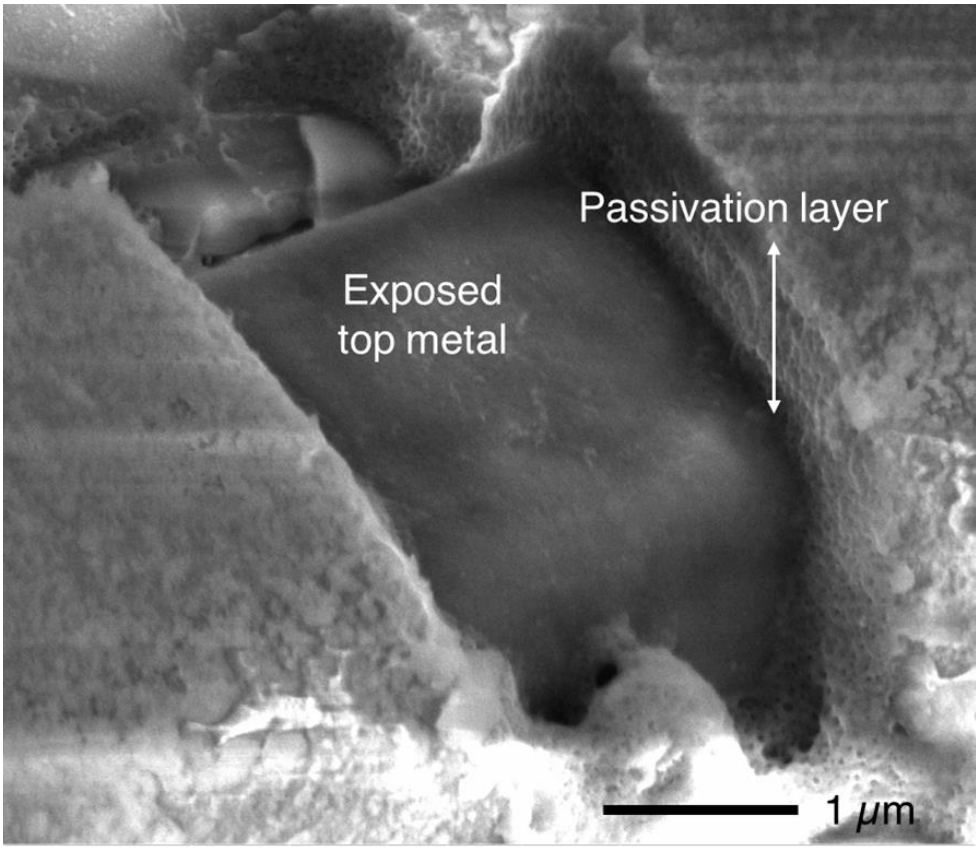

Figure S1. Scanning electron microscope (SEM) image of the top metal fuse after ablating the dielectric passivation layer. The SEM image shows the top metal fuse exposed following the precise removal of the approximately 1 μm-thick dielectric passivation layer (silicon nitride and oxide). The passivation layer was etched using a UV laser pulse with a wavelength of 355 nm and an average power of 0.25 mJ, here after five repetitions.

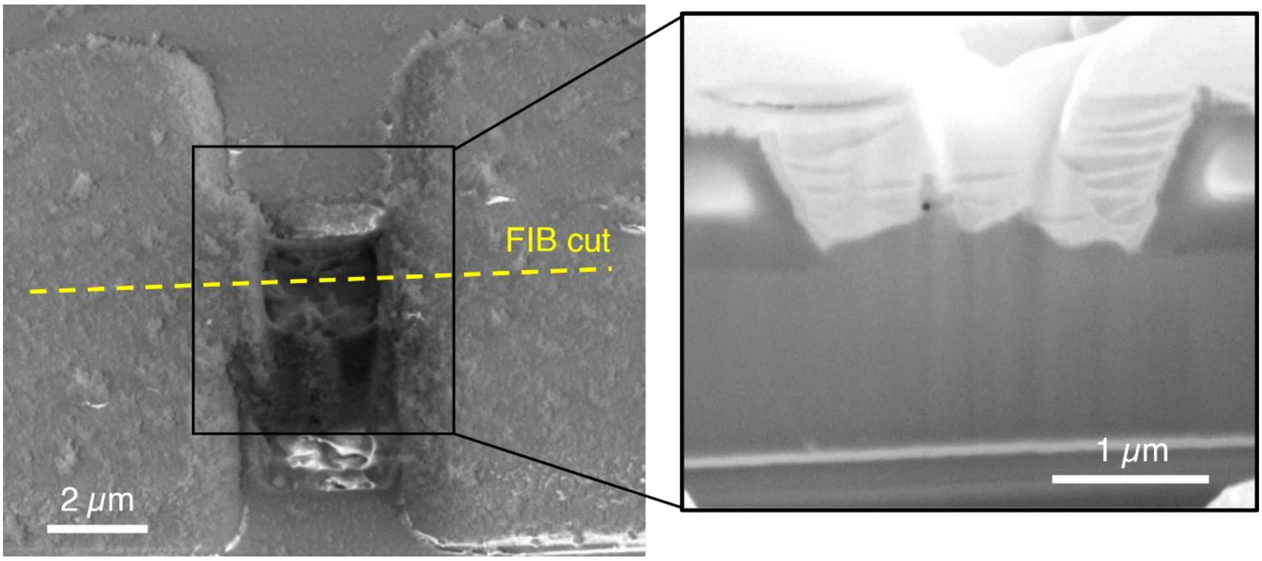

Figure S2. SEM image and FIB cross-section image of the ablated top metal fuse. The SEM image on the left shows a top view of the ablated top metal fuse, with the size of 3 μm × 3 μm, while the image on the right presents a cross-sectional view achieved through focused ion beam (FIB) polishing. The yellow dashed line indicates the FIB cut. Notably, the size of the smallest fuse is restricted to 3 μm × 3 μm, according to the circuit design rule of the chosen process. The image demonstrates the high spatial resolution of the laser ablation technique, allowing for selective removal and cutting of fuse connections while minimizing damage to adjacent circuit structures.

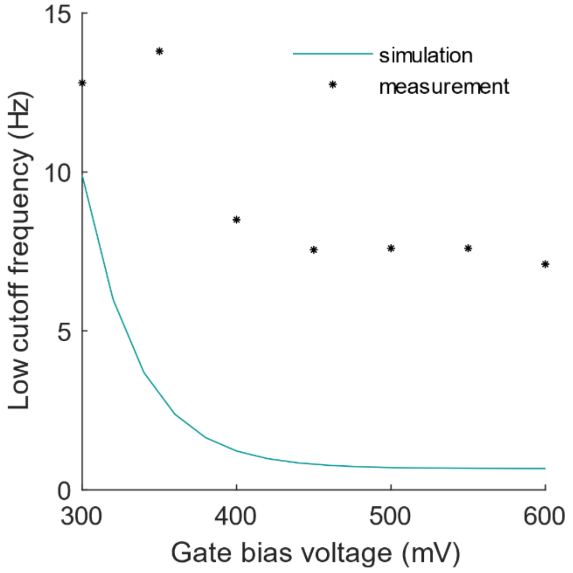

Figure S3. Low cutoff frequency of the single-stage amplifier as a function of the gate bias voltage for the externally biased tunable pseudo-resistors (see main text). Results obtained from simulation and measurement are compared.

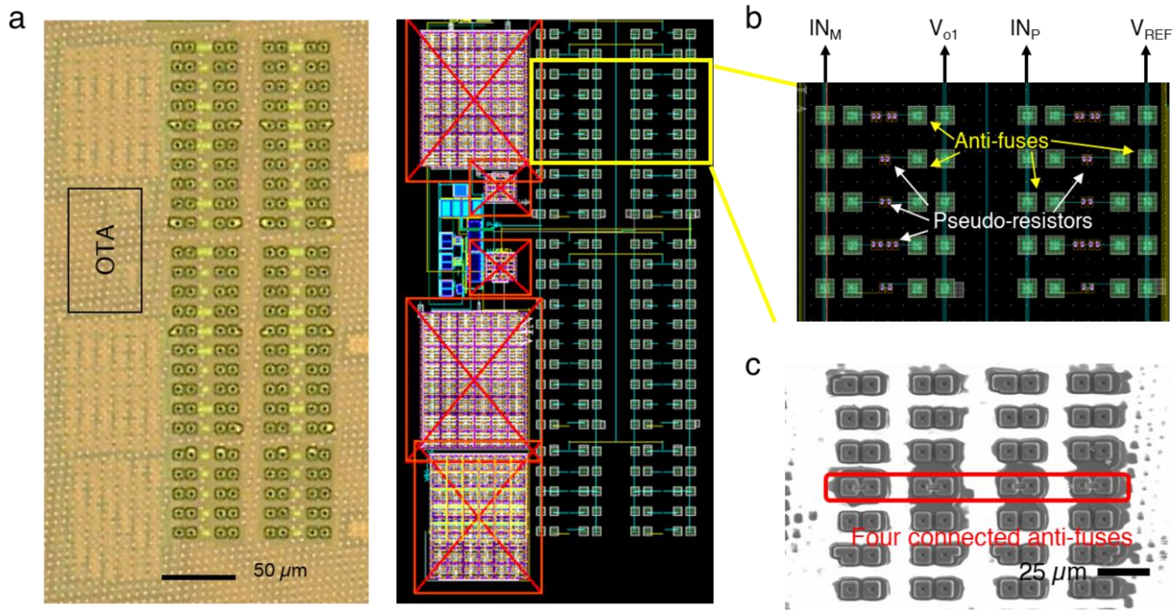

Figure S4. Circuit layout and connection geometry for anti-fuses, pseudo-resistors, and the neural signal amplifier. (a) Circuit photograph depicting 25 types of pseudo-resistors (left), where each pseudo-resistor is connected to a pair of anti-fuses. Circuit layout illustrating the arrangement of the pseudo-resistors and anti-fuses (right). (b) Illustration of the connection scheme between the anti-fuse, pseudo-resistor, and the amplifier shown in Figure 2a. Each anti-fuse comprises two isolated vias (each measuring 6 μm × 6 μm). The passivation layer is selectively removed through FIB milling, enabling Pt interconnect fabrication between the vias using IBID techniques. (c) SEM image showing the alignment of a row of four connected anti-fuses, corresponding to two pseudo-resistors, in the single-stage amplifier experiment.

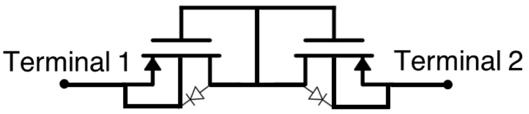

Figure S5. Circuit configuration of a pseudo-resistor evaluated using anti-fuse programming. In contrast to the externally biased pseudo-resistor depicted in Figure 2a, this configuration employs a self-biased pseudo-resistor.

Table S2. Types and parameters for self-biased pseudo-resistors selected by anti-fuse programming

**Label**	**Transistor type**	**Length [μm]**	**Width [μm]**
a	Standard Vt	1	0.2
b	Standard Vt	0.2	0.2
c	Low Vt	0.2	0.2

*Vt: threshold voltage

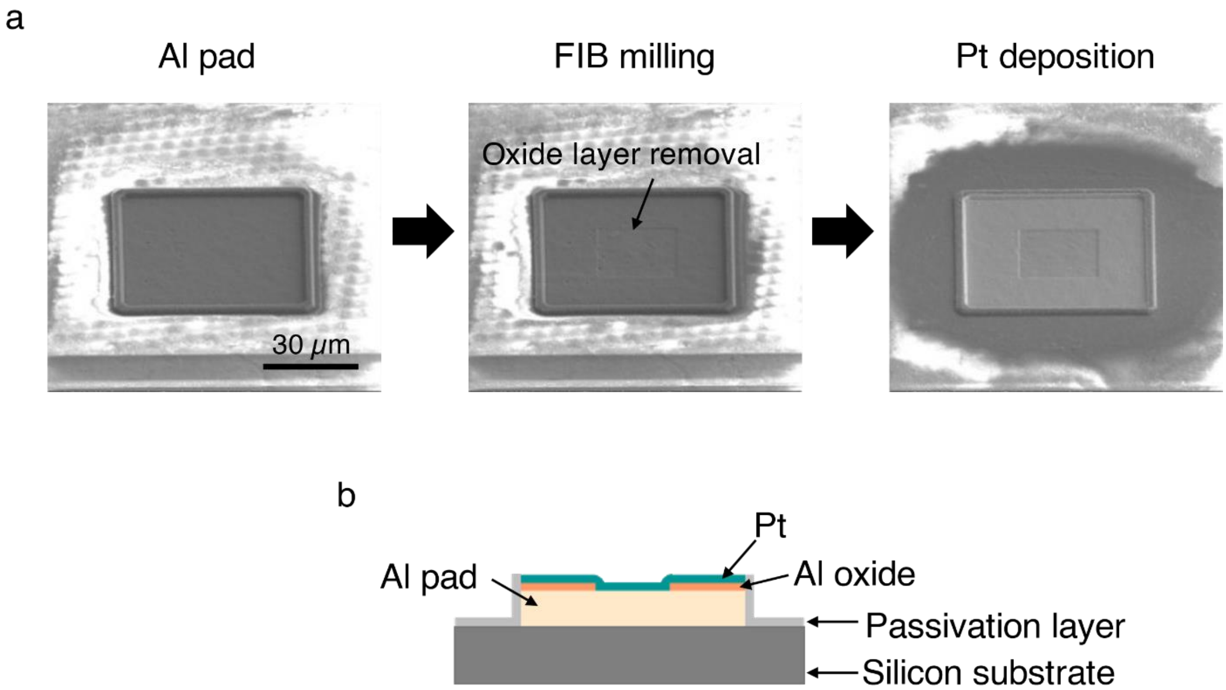

Figure S6. FIB-based on-chip Pt microelectrode fabrication process. (a) FIB milling process was utilized to etch a 500 nm-deep layer, removing the aluminum oxide of the contact pad for access to the bare Al contact pad. A 300 nm-thick layer of platinum was then deposited on top of the Al pad using ion-beam-induced deposition (IBID). (b) Side view schematic illustrating the monolithic integration of the Pt microelectrode on the chip.

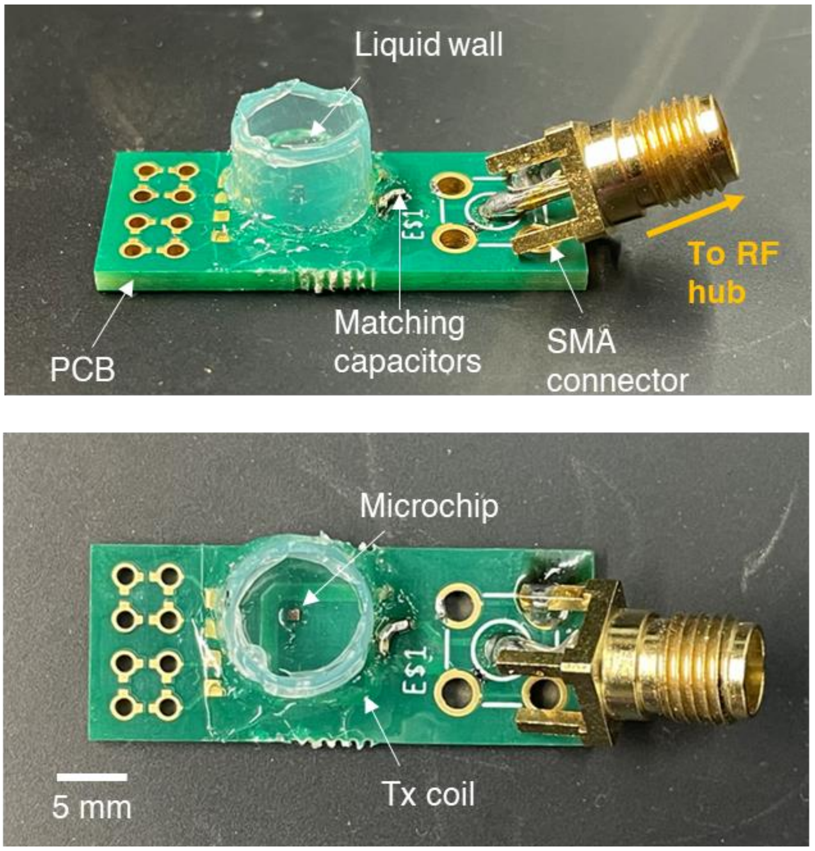

Figure S7. Arrangement for fully wireless chip testing. The image depicts a transmitting (Tx) coil and associated components mounted on an FR4 printed circuit board (PCB) with a saline-holding wall in which the microchips were located. This experimental setup was designed to test chips in a completely wireless environment. During the resonance frequency testing process, the microchip was securely fixed in position, allowing for precise control of the tuning capacitance in a stable electromagnetic environment. To ensure impedance matching, two matching capacitors were utilized to match the impedance of the Tx coil to 50 ohms. The SMA connector was used to connect the Tx coil to the external RF hub and electronics for wireless power transfer and data communication. The SMA connector was tilted to facilitate the laser ablation process, allowing for the objective lens to approach the microchip closely.

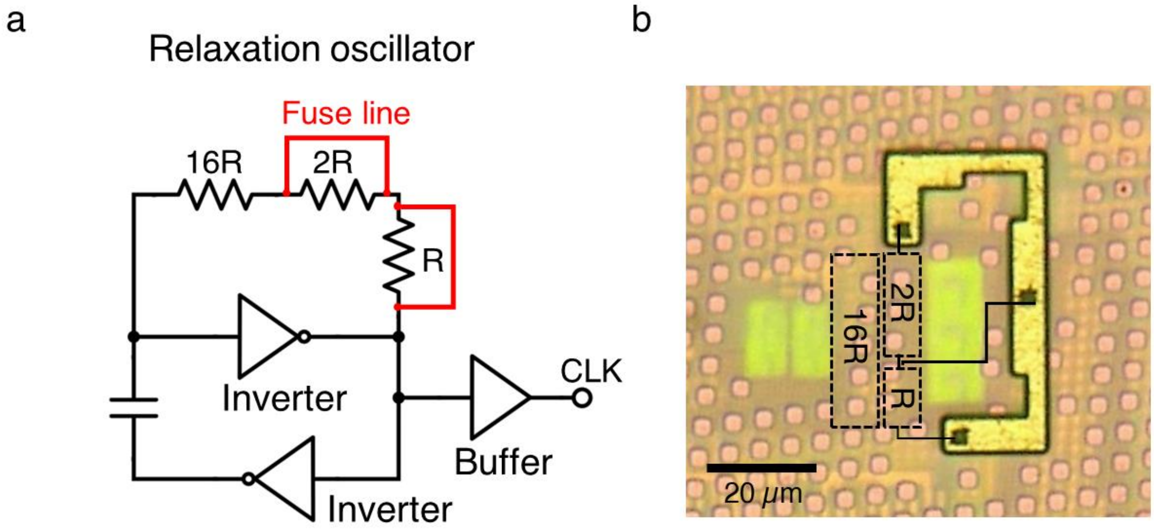

Figure S8. Integration of programmable fuses in relaxation oscillator subcircuits for on-chip clock frequency control. (a) Circuit schematic of the relaxation oscillator utilized for generating a nominally 30 MHz on-chip clock, illustrating the integration of a programmable fuse structure within the oscillator. Cutting the fuse line impacts its time constant, resulting in a decrease in the oscillation frequency. This allows for precise control of the clock frequency within a fixed voltage supply. (b) Microphotograph showing the geometric design of the fuse lines, specifically engineered to avoid RF interference with underlying circuits.

Supplementary References

[1] H. U. Chung, A. Y. Rwei, A. Hourlier-Fargette, S. Xu, K. Lee, E. C. Dunne, Z. Xie, C. Liu, A. Carlini, D. H. Kim, D. Ryu, E. Kulikova, J. Cao, I. C. Odland, K. B. Fields, B. Hopkins, A. Banks, C. Ogle, D. Grande, J. B. Park, J. Kim, M. Irie, H. Jang, J. Lee, Y. Park, J. Kim, H. H. Jo, H. Hahm, R. Avila, Y. Xu, M. Namkoong, J. W. Kwak, E. Suen, M. A. Paulus, R. J. Kim, B. V. Parsons, K. A. Human, S. S. Kim, M. Patel, W. Reuther, H. S. Kim, S. H. Lee, J. D. Leedle, Y. Yun, S. Rigali, T. Son, I. Jung, H. Arafa, V. R. Soundararajan, A. Ollech, A. Shukla, A. Bradley, M. Schau, C. M. Rand, L. E. Marsillio, Z. L. Harris, Y. Huang, A. Hamvas, A. S. Paller, D. E. Weese-Mayer, J. Y. Lee and J. A. Rogers, Nat. Med. 2020, 26, 418–429.

[2] K. Kwon, J. U. Kim, S. M. Won, J. Zhao, R. Avila, H. Wang, K. S. Chun, H. Jang, K. H. Lee, J.-H. Kim, S. Yoo, Y. J. Kang, J. Kim, J. Lim, Y. Park, W. Lu, T.-i. Kim, A. Banks, Y. Huang and J. A. Rogers, Nat. Biomed. Eng. 2023, 1–14.

[3] W. Biedeman, D. J. Yeager, N. Narevsky, A. C. Koralek, J. M. Carmena, E. Alon, and J. M. Rabaey, *IEEE J. Solid-State Circuits,* **2013**, *48*, 960–970

[4] P. Yeon, M. S. Bakir and M. Ghovanloo, 2018 IEEE Cust. Integr. Circuits Conf. (CICC), **2018**, pp. 1–4.

[5] A. Khalifa, Y. Liu, Y. Karimi, Q. Wang, A. Eisape, M. Stana´cevi´c, N. Thakor, Z. Bao and R. Etienne-Cummings, *IEEE Trans. Biomed. Circuits Syst.* **2019**, *13*, 971–985.

[6] J. Lee, V. Leung, A.-H. Lee, J. Huang, P. Asbeck, P. P. Mercier, S. Shellhammer, L. Larson, F. Laiwalla and A. Nurmikko, *Nat. Electron.* **2021**, *4*, 604–614.
